# The role of biological fluid and dynamic flow in the behavior and cellular interactions of gold nanoparticles

**DOI:** 10.1186/s12951-015-0117-1

**Published:** 2015-09-05

**Authors:** Emily K. Breitner, Saber M. Hussain, Kristen K. Comfort

**Affiliations:** Department of Chemical and Materials Engineering, University of Dayton, 524 Kettering Laboratories, 300 College Park, Dayton, OH 45469-0256 USA; Molecular Bioeffects Branch, Bioeffects Division, Human Effectiveness Directorate, Air Force Research Laboratories, 711 HPW/RHDJ, Wright-Patterson AFB, Dayton, OH 45433 USA

**Keywords:** Enhanced in vitro system, Alveolar fluid, Dynamic flow, Gold nanoparticle, Deposition efficiency, Nanoparticle characterization, Nano-cellular interface, Nanoparticle transport

## Abstract

**Background:**

Due to their distinctive physicochemical properties, nanoparticles (NPs) have proven to be extremely advantageous for product and application development, but are also capable of inducing detrimental outcomes in biological systems. Standard in vitro methodologies are currently the primary means for evaluating NP safety, as vast quantities of particles exist that require appraisal. However, cell-based models are plagued by the fact that they are not representative of complex physiological systems. The need for a more accurate exposure model is highlighted by the fact that NP behavior and subsequent bioresponses are highly dependent upon their surroundings. Therefore, standard in vitro models will likely produce inaccurate NP behavioral analyses and erroneous safety results. As such, the goal of this study was to develop an enhanced in vitro model for NP evaluation that retained the advantages of cell culture, but implemented the key physiological variables of accurate biological fluid and dynamic flow.

**Results:**

In this study, a cellular microenvironment was modeled and created after an inhalation exposure scenario. This system comprised of A549 lung epithelial cells, artificial alveolar fluid (AAF), and biologically accurate dynamic flow. Under the influence of microenvironment variables, tannic acid coated gold NPs (AuNPs) displayed modulated physicochemical characteristics, including increased agglomeration, disruption of the spectral signature, and decreased rate of ionic dissolution. Furthermore, AuNP deposition efficiency, internalization patterns, and the nano-cellular interface varied as a function of fluid composition and flow condition. AAF incubation simultaneously influenced both AuNPs and cellular behavior, through excessive NP agglomeration and alteration to A549 morphology. Dynamic flow targeted the nano-cellular interface, with differential responses including modified deposition, internalization patterns, and cellular elongation. Lastly, the biocompatibility of the system was verified to ensure cellular health following AAF exposure and fluid dynamics.

**Conclusions:**

This study confirmed the feasibility of improving standard in vitro models through the incorporation of physiological variables. Utilization of this enhanced system demonstrated that to elucidate true NP behavior and accurately gauge their cellular interactions, assessments should be carried out in a more complex and relevant biological exposure model.

**Electronic supplementary material:**

The online version of this article (doi:10.1186/s12951-015-0117-1) contains supplementary material, which is available to authorized users.

## Background

In recent years, nanoparticle (NP) usage throughout the consumer, industrial, and medical markets has exponentially grown. This is due to the fact that unique properties inherent with nano-sized materials, such as increased transport potential, unique optical properties, and augmented reactivity, make them extremely attractive for product and application development [1]. To date, gold NPs (AuNPs) are among the most frequently investigated and utilized materials. Due to their unique plasmonic properties, general biocompatibility, and ease of functionalization, AuNPs have distinguished themselves as a leading candidate for a vast number of biomedical applications, including photothermal therapy, drug delivery, and enhanced bio-imaging [2, 3]. However, coinciding with this growth in NP utilization is an increased likelihood of unintentional material exposure, with yet unresolved biological consequences.

The rapid development and increased rate of NP exposure has resulted in the critical need to evaluate NP safety, as well as establish recommended exposure limits [4, 5]. This is an arduous task as NPs are variable by nature and include tunable parameters such as size, shape, surface modification, and composition [6, 7]. Current nanotoxicological efforts have identified that both direct and indirect biological impacts, such as cytotoxicity, activation of stress responses, and immune system induction, are dependent on these tunable parameters [8, 9]. These finding, have substantially complicated and hindered efforts to standardize safe NP practices and exposure limits, of which few currently exist [4]. As a result, novel types and classes of NPs are being generated at a pace that far exceeds current capacities for evaluating their safety and establishing occupational protocols.

Currently, NP safety assessments are being carried out in both cell-based in vitro or animal-based in vivo models. As in vitro methodologies provide a fast and cost effective option for NP screening, cell-based systems are predominantly utilized [10]. However, traditional cell culture models possess the considerable drawback that they are not an accurate representation of a physiological environment; thereby producing a less realistic exposure scenario and limited predictive capabilities. While in vivo evaluations can be easily extrapolated to safety guidelines, animal models are encumbered with significant time, regulatory, and financial constraints, limiting their usage. Moreover, due to the innate differences between these models, NP evaluations performed in in vitro systems have demonstrated poor correlation to in vivo analyses [11–13]. As such, there exists a tremendous need for the development of an enhanced, novel cellular system that preserve the advantages of in vitro while incorporating in vivo influences to produce a more realistic and relevant NP exposure scenario.

NPs possess characteristics that distinguish them from traditional chemicals, such as an insoluble nature, agglomeration tendencies, and non-uniform concentration gradients; further complicating the establishment of appropriate exposure conditions [14]. As NP behavior is strongly dependent on environmental factors, recent studies have begun modifying traditional cell culture in an effort to mimic a physiological system and obtain more accurate NP responses. One current approach is to generate an immune inclusive co-culture model, allowing for assessment of immune activation, which is a key biological endpoint frequently overlooked in in vitro investigations [15, 16]. Recent studies identified an active immune response, even in the absence of NP-induced cytotoxicity, demonstrating the importance of including immune functionality and uncovering responses not obtainable with standard techniques. It is also possible to alter the immediate physical environment to greater reflect complex in vivo surroundings. For example, the inclusion of physiological fluids, either artificially synthesized or procured from an in vivo source, replicates an environment that NPs will likely encounter. Current studies using physiological fluids have demonstrated that environmental factors modulate both NP–NP and NP-cell interactions [17–19]. Moreover, the cardiovascular system surrounds all tissues, producing either direct or indirect fluid movement. Current in vitro techniques are static by nature and as such are neglecting this significant physiological influence. Early studies demonstrated that the introduction of lateral flow to a NP system altered the balance between diffusion and sedimentation forces, thereby modifying dosimetry, NP internalization, and bioresponses [20, 21].

Extensive work has been done to develop associations between NP parameters and observed cellular consequences [6, 7]. However, recent investigations have demonstrated that NP physicochemical properties are altered by physiological variables [17–21]: providing a rationale for the poor correlation between in vitro and in vivo NP assessments. Taken together, these facts suggest that to improve predictive capabilities for NP safety evaluations and efficacy of nano-based applications, an enhanced exposure system is required [22]. As previously discussed, in vitro systems have been successful modified through a number of means, providing a mechanism to develop biologically accurate evaluation of NP behavior and induced biorepsones.

It is established that the extent and mechanism of NP interaction with the cellular membrane, known as the nano-cellular interface, determine observed cellular responses. These evaluations are challenging to perform in vitro [23] and nearly impossible in vivo. To overcome these challenges, recent advances have been made in the predictive modeling of AuNP interactions with lipid membrane and their degree of endocytosis following exposure [24–26]. These studies have successfully identified the capabilities of AuNPs to translocate into cytoplasm and correlate uptake kinetics to cytotoxicity. Given both the high number of NPs that require testing and the novel conditions arising from enhanced in vitro systems, these simulations hold tremendous potential to predict NP internalization patterns and bioresponses.

The goal of this study was to design and implement an enhanced cellular microenvironment for improved assessment of NP behavior. As inhalation is a common route of NP exposure, our generated microenvironment was designed as an alveolar system and incorporated a lung alveolar epithelial cell line (A549), artificial alveolar fluid (AAF), and dynamic flow at accurate physiological rates. Following introduction of tannic acid coated AuNPs to the microenvironment, our results demonstrated modified AuNP behavior and reshaping of the nano-cellular interface. Taken together, these results indicate that enhanced in vitro models, can uncover novel responses not present in a traditional in vitro model, and may be better suited for evaluation of nano-based products and applications.

## Results and discussion

### Generation of enhanced cellular microenvironment

In an effort to more accurately mimic in vivo NP exposure, the microenvironment generated in this study incorporated three critical design elements: physiologically accurate flow rates, biological fluids that NPs would likely encounter, and a cell model appropriate for the selected fluid. Dynamic flow was established through the use of a multi-channel peristaltic pump, which allowed for the simultaneous evaluation of different samples. The pump operated at a target volumetric flow rate of 0.75 mL/min, which produced an average linear velocity of 0.63 cm/s with the tubing; matching standard velocities of the cardiovascular system [27]. The A549 cells experienced a fluid velocity of 0.0065 cm/s across their surface, which in addition to being orders of magnitude lower than the tubing, is representative of the mass and fluid transport occurring between the alveolar cells and surrounding capillary networks [28].

**Fig. 1:**
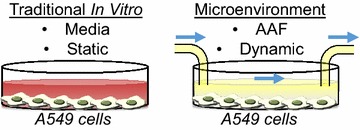
Comparison of a traditional in vitro model to the microenvironment utilized in this study. Primary differences that exist between these models was replacing media with AAF and the introduction of dynamic flow. All experimentation was carried out in 24-well plates

One major advantage of the current microenvironment design is that it can be customized to target specific cellular locations, simply by selecting relevant cell types, incorporating a corresponding fluid, and adjusting the flow to predetermined rates. For this proof-of-concept study, an alveolar lung region was modeled, as inhalation is a primary route of NP exposure [29]. The A549 model, an alveolar basal epithelial cell line, was specifically chosen due to its relevance, robustness, and the fact that it has been extensively studied following NP introduction [30]. To support the lung exposure route, artificial alveolar fluid (AAF) was implemented, as this fluid is the predominant environment in the alveolar region of the lungs. During evaluation, a matrix of static/dynamic and media/AAF conditions were generated and compared to identify the role of each variable on NP characterization, cellular responses, and the nano-cellular interface. A schematic of microenvironment design is shown in Fig. 1.

### NP selection and initial characterization

This study utilized tannic acid coated, 60 nm AuNPs as the experimental target within the generated microenvironment system. AuNPs were included in this study owing to the considerations that they are one of the mostly commonly employed NPs, their synthesis procedures are optimized, and their biologically responses are well documented; providing both a rationale for their investigation and benchmark behavioral responses [3, 8]. Furthermore, AuNPs are being explored for drug delivery and imaging techniques within the lungs, making them relevant to our target system [31]. As surface chemistry has been proven to dictate cellular interactions, and thereby responses, tannic acid was specifically chosen owing to its known ability to promote NP–NP interactions as well as its documented protein affinity [23, 32].

**Fig. 2:**
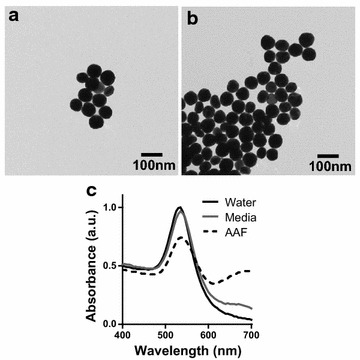
Characterization of the 60 nm tannic acid AuNPs. **a** Representative TEM image of the stock AuNPs verified spherical morphology and was used for analysis of primary NP size. **b** TEM imaging of the AuNPs was repeated after a 24 h incubation in AAF. **c** Spectral profile of the AuNPs following dispersion in water, media, and AAF demonstrate a fluid-specific disruption of the AuNPs plasmonic properties

As the field of nanotechnology has developed, the vital need for NP characterization has emerged. Therefore, prior to cellular exposure, evaluation of key NP physicochemical properties was carried out through the standard array of characterization techniques. Transmission electron microscopy (TEM) confirmed spherical particle morphology and was used to determine the primary particle size of 65 nm (Fig. 2a). Within a fluid environment, all NPs will agglomerate to some degree, although final aggregate size is dependent on both NP physicochemical parameters and environmental composition [17, 32]. In water, the AuNPs displayed minimal inter-particle binding with a final agglomerate size of approximately 75 nm (Table 1). Through zeta potential analysis, it was determined that stock AuNP displayed a negative charge, in accordance with the tannic acid coating. Lastly, the rate of ionic dissolution was determined after a 24 h incubation in water, and found to be a marginal 0.8 %.

**Table 1:** Characterization of the stock AuNPs

Primary size (nm)	65.1 ± 5.3
Agglomerate size (nm)	74.8 ± 4.6
Zeta potential (mV)	−31.8 ± 0.9
Ionic dissolution (%)	0.8 ± 0.5

### Fluid-induced alterations to AuNP characteristics

Recent studies have elucidated an intrinsic link between local environmental factors, NP behavior, and resultant cellular effects [8, 9]. Therefore, we wanted to isolate and identify if NP behavior within the microenvironment was a function of both dynamic flow and fluid composition prior to cellular exposure. Initial characterization efforts centered on agglomeration tendencies, spectral profiles, and kinetic rates of ionic dissolution in an acellular environment.

Following incubation in AAF, the AuNPs underwent TEM imaging to identify if basic morphology or primary size was altered (Fig. 2b). From this image, it was seen that the AuNPs were still spherical in nature with no change to primary diameter. Next, AuNP spectral profiles were generated following dispersion in water, media, and AAF (Fig. 2c). In water, the profile demonstrated a single, well defined plasmonic peak at approximately 550 nm, confirming the quality and uniformity of the NP set. In media, the spectral signature was relatively unchanged, with a minor right-shift identified. However, in AAF the AuNPs exhibited a significant loss in maximum absorbance, accompanied by extensive peak broadening and the appearance of a second peak at approximately 700 nm. The presence of additional peaks are indicative of excessive NP agglomeration, whereas peak broadening and decrease in absorbance are associated with loss of stability and particle sedimentation [33]. Therefore, the spectral profiles suggest that distribution in AAF induced significant modifications to AuNP characteristics.

**Fig. 3:**
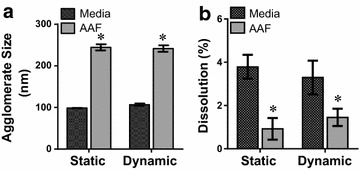
AuNP Characterization as a function of fluid and flow. Evaluation was carried out to identify if **a** AuNP agglomerate size and **b** rate of ionic dissolution varied under the influence of microenvironment variables (data represents four independent trials, *asterisk* denotes statistical significance between media and AAF samples under the same flow condition)

To confirm this theory, the extent of NP agglomeration in media and AAF was evaluated via dynamic light scattering (DLS) (Fig. 3a), and agreed with the previous spectral profiles. A minor increase in agglomeration was noted for media, corresponding with the slight right shift in the plasmonic peak. In contrast, AAF exposure resulted in considerable AuNP agglomeration, with final aggregate sizes of approximately 260 nm. Therefore, it is probable that these large NP agglomerates became unstable and altered the spectral profile. Moreover, the fluid specific AuNP agglomeration patterns were further verified via inspection: with a visible color shift from red to purple in AAF (Additional file 1: Figure S1). Taken together, these results demonstrated that degree of NP agglomeration, a critical behavior, was dependent upon environmental composition.

The next step was to ascertain if exposure to dynamic flow modified AuNP agglomeration. As seen in Fig. 3a, irrespective of fluid composition, the introduction of shear stress through dynamic flow had no impact on agglomerate size. As the flow rate in this study was set to low, biological levels, these results were not surprising. To further explore the stability of these aggregates, this experiment was repeated at a significantly higher flow rate, with a tube-side velocity of 5.5 cm/s (Additional file 1: Figure S2). Even following 24 h exposure to high flow and shear stress rates, NP agglomeration remained unaltered.

Lastly, the rate of AuNP ionic dissolution was evaluated as a function of environmental composition and dynamic flow (Fig. 3b). While ionic dissolution may not be a standard characterization assessment for AuNPs, it is a critical element for a variety of other NPs, including silver, titanium dioxide, and copper [34]. As such, we determined if and to what degree ion generation varied in the microenvironment. While dissolution rates between static and dynamic conditions were unvaried, a fluid dependency existed, with AAF exposure decreasing ion production. This fluid-specific effect agrees with previous literature that correlated higher dissolution rates to smaller NP agglomerates [35]. Due to extensive AuNP agglomeration in AAF, they exhibited a smaller surface area to volume ratio, thus resulting in lower NP reactivity and ion production.

### Biocompatibility of the AuNPs and the microenvironment

AuNPs are renowned for their biocompatibility, which is one feature that makes them attractive for nano-based applications. However, as the AuNPs displayed modified properties under the influence of microenvironment variables, we wanted to ensure that no negative synergistic biological responses occurred. Biocompatibility within the microenvironment was confirmed through quantification of lactate dehydrogenase (LDH) leakage (cytotoxicity) and reactive oxygen species (ROS) production (oxidative stress).

**Fig. 4:**
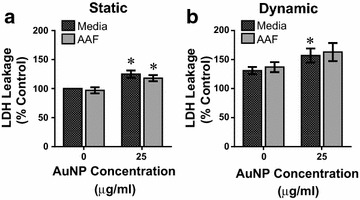
LDH release from A549 cells under microenvironment influences. **a** Evaluation of LDH release in a static environment after a 24 h exposure to the denoted conditions. **b** Toxicological analysis under dynamic conditions demonstrated greater LDH secretion. For both static and dynamic, control conditions were static/media without AuNPs (data represents three independent trials, *asterisk* denotes statistical significance between 0 and 25 μg/mL AuNPs in the same fluid/flow conditions)

The results from the LDH analysis are shown in Fig. 4 and revealed an increase in LDH release as a function of AuNP dosage and flow, but not fluid composition. Looking first at static conditions (Fig. 4a), AAF and media alone had comparable LDH levels, indicating that AAF exposure didn’t induce apoptosis. Following the addition of AuNPs, a minimal increase in LDH was observed for both fluids, as anticipated, but not to a degree indicative of cell death. Moving to dynamic conditions (Fig. 4b), dynamic flow increased LDH levels over static, but no synergistic response occurred. Again there was a minimal increase in LDH following the addition of AuNPs, but as before, still within biocompatible regimes.

**Fig. 5:**
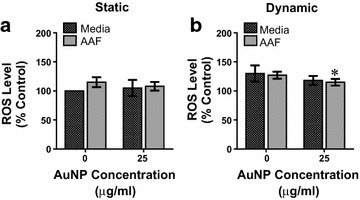
Evaluation of ROS production following AuNP exposure within the microenvironment. **a** ROS generation was evaluated under static conditions to the denoted exposure parameters. **b** ROS levels were quantified under dynamic conditions for AuNPs in either media or AAF. For both static and dynamic, control conditions A549 cells under static/media conditions without AuNPs (data represents three independent trials, *asterisk* denotes statistical significance between 0 and 25 μg/mL AuNPs in the same fluid/flow conditions)

Next, stress activation was evaluated through ROS production (Fig. 5). ROS was specifically selected as it is a recognized marker of cellular stress and serves as an early predictor of apoptosis [36]. Analogous to LDH release, no fluid-specific effect was observed, but ROS was a function of both AuNP addition and flow status. Under static conditions (Fig. 5a), ROS generation was essentially equivalent for all examined parameters, confirming the safety of AAF and flow exposure. When examining dynamic results (Fig. 5b), basal ROS levels were increased approximately 30 % by the presence of fluid flow. As fluid-induced shear stress is known to cause basal ROS production, the increase over static conditions was not surprising [37]. Interestingly, following AuNP exposure under dynamic conditions, the ROS levels decreased, likely due to the ability of AuNPs to act as an antioxidant and counteract the increased ROS levels [38]. Therefore, we confirmed that exposure to dynamic flow and AAF within the microenvironment, both with and without the addition of AuNPs, did not elicit an overtly harmful biological response.

### Modulation of AuNP deposition and the nano-cellular interface

**Fig. 6:**
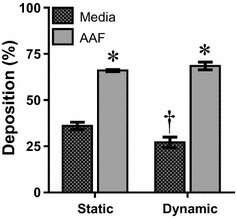
AuNP deposition efficiency associated with A549 cells. Evaluation of NP deposition after a 24 h exposure was carried out in both media and AAF environments under static and dynamic flow conditions. (data represents four independent trials, *asterisk* denotes statistical significance between media and AAF samples under the same flow condition, *dragger* denotes significance between static and dynamic flow conditions for the same fluid environment)

Following evaluation of NP physicochemical properties and assessment of biocompatibility under microenvironment influences, we next examined the nano-cellular interface. The first means of assessment was to determine the fraction of NPs that associated with the cell surface, referred to as the NP deposition efficiency (Fig. 6). Looking at fluid comparisons, incubation in AAF more than doubled the deposited AuNP dose over media conditions. This is due to the extensive AuNP agglomeration in AAF, shifting predominant transport mechanisms from diffusion to sedimentation [39, 40].

The introduction of dynamic flow altered deposition efficiency, in conjunction with environmental factors. For AAF exposure no change was noted between static and dynamic, again due to the presence of large agglomerates, strong sedimentation forces, and the ability of tannic acid to tightly couple with the protein membrane. However, for media, a drop in deposition occurred following the introduction of fluid flow. This loss is due to the forced lateral movement of the system, which overpowers and disrupts the diffusion rate, thus inhibiting contact between AuNPs and A549 surfaces [41].

**Fig. 7:**
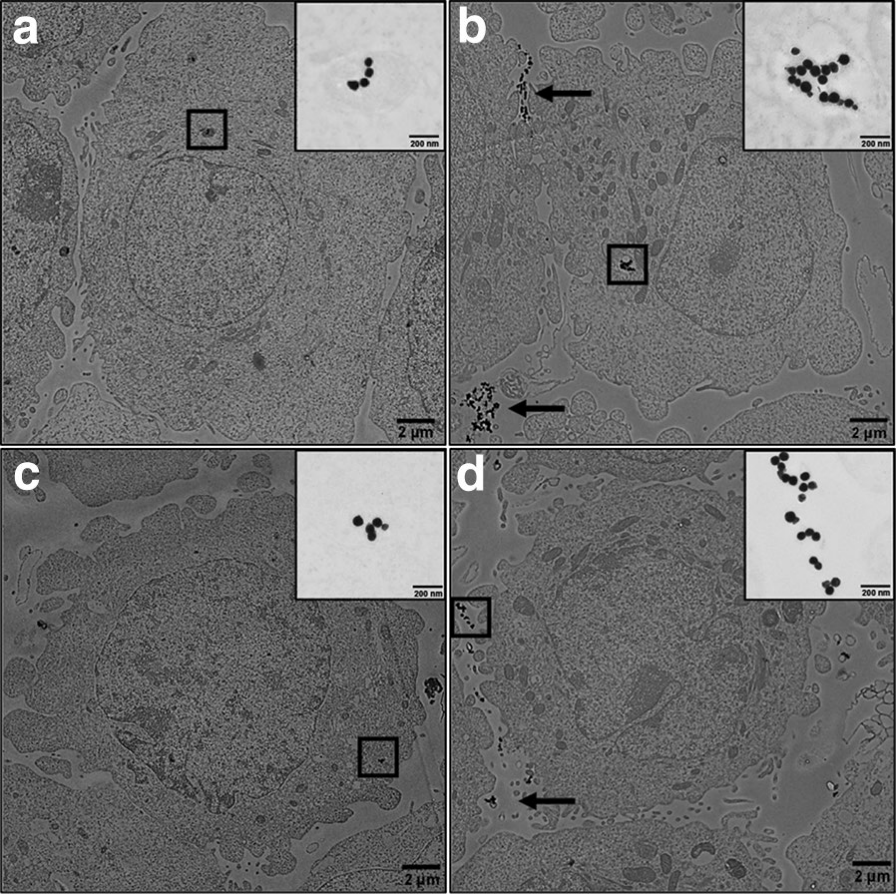
TEM visualization of AuNP deposition patterns. Representative images are shown of A549 exposure under the following conditions: **a** static media, **b** static AAF, **c** dynamic media, and **d** dynamic AAF. The *area within the black box* is enlarged within the inset for each condition. *Black arrows* point out additional AuNP agglomerates bound to the cell surface, but not internalized

Investigation into the deposition of the AuNPs was taken one step further and TEM images were obtained to visualize uptake patterns within the A549 model (Fig. 7). Control images of A549 cells in media and AAF under static and dynamic conditions are included in Additional file 1: Figure S3. Looking first at media (Fig. 7a, c), small AuNP agglomerates are found internalized within the cytoplasm. Furthermore, no discernable differences were noted under static versus dynamic conditions, in general agreement with the deposition data.

When looking at AAF (Fig. 7b, d), TEM imagery confirmed greater deposition, with large particle clusters and NP-cell associations easily visible. In static conditions, AAF aggregates were located both within intracellular vacuoles and bound to the cell surface (denoted by arrows). Interestingly, under dynamic flow in AAF, no internalized AuNPs were identified, with all particles remaining bound to the outside cell membrane. Therefore, while deposited dose was unaltered, the effective internalization rate was a function of flow condition. Taken together, these TEM images confirm that both fluid environment and flow status impact the AuNP deposition as well as internalization patterns.

**Fig. 8:**
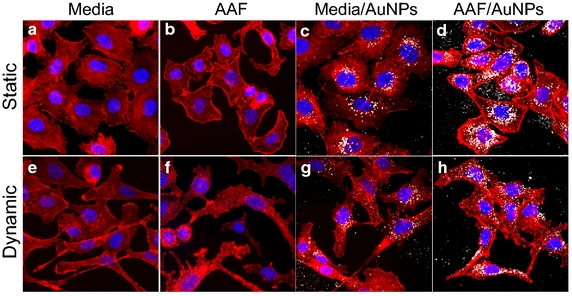
Visualization of the nano-cellular interface within the microenvironment. Representative images are shown for: **a** static media control, **b** static AAF control, **c** static media with AuNPs, **d** static AAF with AuNPs, **e** dynamic media control, **f** dynamic AAF control, **g** dynamic media with AuNPs, and **h** dynamic AAF with AuNPs. Directionality of flow was from* left* to* right* across the image. In these images, actin and nuclei are stained *red* and *blue*, respectively, with the NPs appearing as *white*

Finally, the nano-cellular interface was examined using high resolution fluorescence microscopy (Fig. 8), revealing much regarding cellular behavior and modes of NP/A549 interactions. Firstly when examining AuNP exposure in media (Fig. 8c, g), fewer particles were seen than with AAF (Fig. 8d, h), in agreement with deposition results. When in an AAF environment, the visible AuNPs were larger and denser, consistent with TEM images. Of greater interest, however, are the extensive influences that the microenvironment had on cellular morphology. Even under the low flow rates, cellular elongation in the directionality of flow was identified. Moreover, incubation in AAF produced a curved cell morphology (Fig. 4d), which has been reported in in vivo alveolar epithelial cells as a result of the high surface tension associated with alveolar fluid [42]. Both these observations were verified with standard light microscopy (Additional file 1: Figure S4) to ensure that this was not an artifact of fixing the cells, but present in live cultures as well. Taken together, these results confirm that both fluid composition and dynamic flow modified the nano-cellular interface.

### Implications of these results

Through the inclusion of relevant physiological variables, we successfully generated an enhanced in vitro system more closely represented in vivo models. This system was designed to target an alveolar NP exposure and included alveolar epithelial cells, artificial alveolar fluid, and low dynamic flow representative of accurate biotransport rates. As it is well established that NP characteristics and behavior are dependent on both key physicochemical traits and the surrounding environment, we anticipated that the 60 nm tannic acid coated AuNPs would exhibit modified traits.

Starting with behavior of the AuNPs acellularly, an AAF environment resulted in significant AuNP agglomeration and dependent spectral shifts. The AAF aggregates were approximately 2.5 times larger, likely leading to a loss of particle stability and increased sedimentation [39, 40]. Due to the large effective size, AuNPs in AAF had a lower surface area to volume ratio versus their media counterpart, resulting in a significantly lower ionic dissolution rate [35]. While ion formation is not critical for AuNPs, the generation of ions from other metallic NPs, such as silver, titianium dioxide, and copper, have been directly correlated to cytotoxicity and disruption of cellular homeostasis, making it an important behavioral endpoint [34, 43]. These results suggest that if other NPs follow similar agglomeration patterns, ion-dependent cytotoxicity may less influential following inhalation exposure.

The nano-cellular interface was also transformed following exposure in the microenvironment. The aforementioned AAF-induced agglomeration and increased sedimentation also resulted in a substantially greater deposited dose. Increased deposition in AAF over media was confirmed both with TEM and fluorescence imaging. The introduction of fluid dynamics also impacted both deposition and the nano-cellular interface. In media, the introduction of lateral flow was able to reduce AuNP deposition, likely through disruption of established diffusion patterns. This has been previously seen in literature, with dynamic flow reducing the contact between NPs and surrounding biological systems [39–41].

In conjunction with AAF, dynamic flow impacted the effectiveness of cellular internalization. Under static flow, TEM images demonstrate both AuNP internalization and surface association. However, for AAF/dynamic environments, no evidence of internalization was seen; AuNPs were only identified coupled to the plasma membrane. An explanation for this may arise from the fluorescence imaging which highlighted significant morphological alterations. Dynamic flow induced cellular elongation and AAF exposure promoted membrane curvature. While these morphological phenomena are found in literature [42] we hypothesize that this fluid-specific alteration is a result of the phosphatidylcholine additive of AAF; a lipid which is a major component of the cellular membrane. Excess phosphatidylcholine has been associated with loss of membrane integrity, supporting this supposition and agreeing with the observed membrane curvature [44]. Given both the simultaneous elongation and membrane alterations under concurrent AAF/dynamic exposure, it is possible that standard mechanisms of cellular internalization, which are initiated at the cell surface, are inhibited as a result of these cellular disruptions.

## Conclusions

The goal of this study was to construct an enhanced in vitro model that incorporated physiological fluids and dynamic flow in an effort to generate a means of rapid NP evaluation in a more relevant model. Through the selection of an alveolar cell line (A549) and use of AAF, our devised microenvironment targeted an alveolar NP exposure scenario. Through changes to morphology that align with in vivo, including cellular elongation and curved membranes, this study demonstrated that inclusion of dynamic flow and biological fluids are positive steps towards increasing in vitro model relevance. Additionally, we identified modified NP behavior, confirmed A549 viability, and observed alteration of the nano-cellular interface. Moreover, a number of these responses were undetected in a traditional in vitro model, supporting the emerging conviction that environmental variables are capable of influencing NP-dependent bioeffects. As such, the utilization of a complex biological model, such as the one devised and implemented here, is necessary to accurately and effectively evaluate the safety and performance of NPs and nano-based applications.

## Methods

### Cell culture

The A549 human alveolar epithelial cell line was purchased from ATCC (American Type Cell Culture) and grown in RPMI 1640 medium, supplemented with 1 % penicillin/streptomycin and 10 % fetal bovine serum. Cultures were maintained on tissue culture treated petri dishes (BD Falcon) in a 5 % CO_2_ incubator at 37 °C. For experimentation, A549 cells were seeded at a density of 1.6 × 10^5^ cells per well in a 24-well plate and returned to the incubator for 24 h to equilibrate.

### Dynamic Flow Exposure System

Dynamic flow was implemented using a multi-channel peristaltic pump (Ismatec, model #ISM939D) with each channel exclusively connected to a single well (1.56 cm diameter) of a 24-well plate. The 1/16 inch (inner diameter) tubing was secured through the culture plate lid to ensure unidirectional flow. Prior to exposure, the tubing was primed to ensure consistent liquid heights between static and dynamic conditions. The entire pump system was stored within the incubator to maintain environmental conditions. During experimentation, flow was set to a target volumetric flow rate of 0.75 mL/min, producing average linear velocities of 0.63 and 0.0065 cm/s in the tubing and across the A549 surface, respectively. This flowrate was specifically chosen to match physiological patterns, with the NPs experiencing equivalent cardiovascular-based flow in the tubing and the cells undergoing flow comparable to diffusion and facilitated transport rates [27].

### Artificial alveolar fluid

Artificial alveolar fluid (AAF) served as the physiologically relevant environment in this study. Alveolar fluid exists in the pulmonary alveolus, forming a coating over the cells that comprise the alveolar wall, and facilitates transport between air and the surrounding capillaries. AAF was synthesized using previously published recipe by Stopford et al. [45]. AAF was comprised of numerous salts supplemented with the lipid phosphatidylcholine with a pH of 7.4.

### Nanoparticle characterization

The 60 nm, tannic acid AuNPs were purchased from nanoComposix as a concentrated liquid stock. For all experimentation, NP solutions were freshly prepared by diluting the stock to a final concentration of 25 µg/mL in the designated fluid. TEM was carried out on a Hitachi H-7600 microscope to visualize the AuNPs. NP agglomeration and surface charge was measured through DLS and zeta potential analyses, respectively, using a Malvern Zetasizer Nano ZS. The NP spectral profile was visualized with UV–VIS using a SpectraMAX Plus 190 microplate reader.

For evaluation of NP ionic dissolution, the area under the spectra curve was measured before and after a 24 h incubation, under the denoted conditions. Standard curves were generated for each NP/fluid combination to directly correlate area under the curve to NP concentration. Dissolution was calculated by determining percent mass lost during incubation.

### Biological response evaluation

LDH release was used to assess cytotoxicity. A549 cells were plated in 24-well plate at density of 1.6 × 10^5^ cells per well, allowed time to adhere, then underwent exposure to the denoted conditions for 24 h. The quantity of LDH released was quantified using the CytoTox 96 Non-Radioactive Cytotoxicity Assay (Promega) in accordance with the manufacturer’s instructions. Additional fluid specific controls were added to account for the change in media and AAF color, as this assay has a colorimetric endpoint.

Production of ROS was used to assess A549 stress activation. Again, A549 cells were plated in 24-well plates and equilibrated. The cultures were then treated with 100 μM of dichloroofluorescein diacetate (DCFH-DA) for 30 min, washed, and exposed to the denoted conditions. After 24 h, the fluorescence was measured using a Spectra MAX Gemini Plus fluorescent plate reader, in accordance with the manufacturer’s instructions.

### NP deposition efficiency

For NP deposition, 1.6 × 10^5^ cells were plated per well in a 24-well plate. Following a 24 h growth period, the A549 s were washed and replenished with the indicated fluid/NP combination (25 μg/mL) for a 24 h exposure, under either static or dynamic conditions. Fluid samples were then removed without disturbing the cells, and the final NP concentration determined through UV–VIS analysis [46]. The change in NP solution concentration following cellular exposure was used to calculate the deposition efficiency. Fluid samples were also removed after 30 min to account for any non-specific binding of NPs to the tubing or culture dishes.

### Visualizing NP uptake

For NP uptake, 1.6 × 10^5^ cells were plated per well in a 24-well plate and equilibrated, then exposed to the indicated NP/fluid combination (25 µg/mL) in either static or dynamic flow conditions for 24 h. The cells were then washed, pelleted, fixed in a 2 % glutaraldehyde/2 % paraformaldehyde solution (Electron Microscope Sciences; EMS), stained with 1 % ssmium tetroxide (EMS), and dehydrated in increasing ethanol concentrations. Cell pellets were then cured in LR white resin (EMS) overnight in a vacuum oven. Cell pellets were thinly sectioned using an ultramicrotome (Model EM UC7, Leica,) and imaged via TEM.

### Evaluating the nano-cellular interface

For cellular morphology evaluation, 1.5 × 10^5^ cells were plated per chamber on a 2-well chambered slide and returned to the incubator to adhere. The cells were then exposed to the NP/fluid combinations in either static or dynamic flow conditions for 24 h. Following exposure, the cells were fixed with 4 % paraformaldehyde and incubated with Alexa Fluor 555-phalloidin (Invitrogen) for actin staining and 4′,6-diamidino-2-phenylindole (DAPI; Invitrogen) for nuclear staining. The slides were then sealed and imaged using a CytoViva 150 ultraresolution attachment and an Olympus BX41 microscope (Aetos Technologies). Images were captured and compiled using QCapture Pro Imaging Software.

### Statistical analysis

Data is expressed as the mean ± the standard error of the mean (SEM). A two-way ANOVA with a Bonferroni post-test was performed on data sets using Graph Pad Prism to identify statistical significance between flow conditions and fluid environments, as indicated. In all cases, a *p* value threshold of 0.05 was set for significance.

## Additional file

Additional file 1:
**Figure S1.** Visual verification of AuNP agglomeration. **Figure S2.** AuNP agglomeration under varying flow rates. **Figure S3.** Control TEM images. **Figure S4.** Light microscopy verification of cell morphology.

## Abbreviations

NP: nanoparticle
AuNP: gold nanoparticle
AAF: artificial alveolar fluid
TEM: transmission electron microscopy
DLS: dynamic light scattering
LDH: lactate dehydrogenase
ROS: reactive oxygen species
