# Supplementary material for: The role of biological fluid and dynamic flow in the behavior and cellular interactions of gold nanoparticles
Source: J Nanobiotechnology. 2015 Sep 5;13:56. doi: 10.1186/s12951-015-0117-1 (PMC4560888; doi:10.1186/s12951-015-0117-1)
Supplement: Additional file 1: — Figure S1. Visual verification of AuNP agglomeration. Figure S2. AuNP agglomeration under varying flow rates. Figure S3. Control TEM images. Figure S4. Light microscopy verification of cell morphology. [file 12951_2015_117_MOESM1_ESM.docx]

The Role of Biological Fluid and Dynamic Flow in the Behavior and Cellular Interactions of Gold Nanoparticles

*Emily K. Breitner^1^, Saber M. Hussain^2^, Kristen K. Comfort^1*^*

^1^Department of Chemical and Materials Engineering, University of Dayton, Dayton, OH USA

^2^Molecular Bioeffects Branch, Human Effectiveness Directorate, Air Force Research Laboratories, Wright-Patterson AFB, OH USA

**Supplemental Figures:**


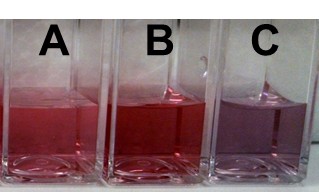


**Supplemental Figure 1:** Visible inspection of AuNP agglomeration patterns following dispersion in (A) water, (B) media, and (C) AAF. The color shift from red to purple in AAF indicates extensive particle agglomeration and is supported by the shift in the spectral profile.

**Supplemental Figure 2:** Influence of flow rate on AuNP agglomeration. DLS analysis was carried out following a 24 hour exposure of AuNPs under high (5.5 cm/s) and low (0.63 cm/s) linear velocities. This data demonstrated negligible changes to agglomerate size with increased flow rate.


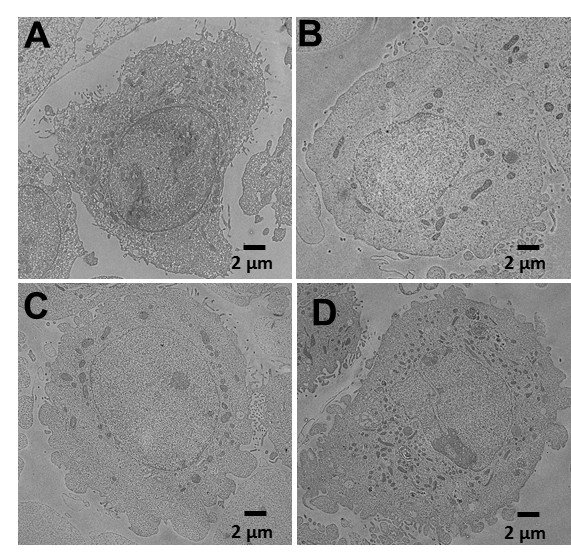


**Supplemental Figure 3:** Representative control images for TEM evaluation of A549 cells in the following environments: (A) static media, (B) static AAF, (C) dynamic media, and (D) dynamic AAF.


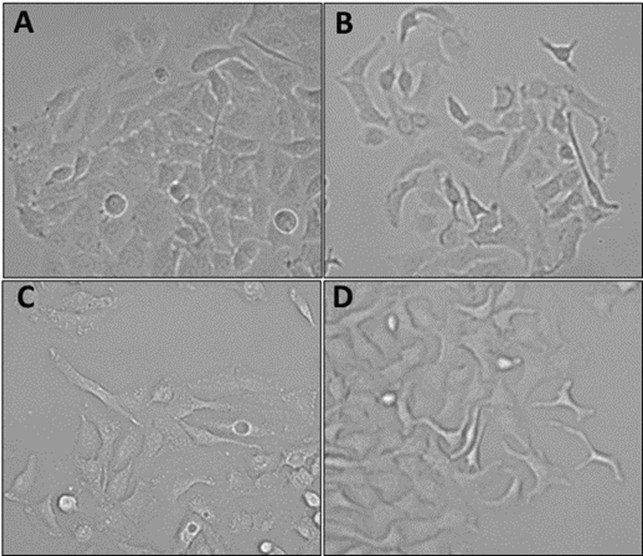


**Supplemental Figure 4:** Light microscopy images were captured of A549 cells in the following environments to verify fluid and flow dependent alterations to cellular morphology: (A) static media, (B) static AAF, (C) dynamic media, and (D) dynamic AAF.
